# Pyruvate Kinase Is Required for Sex Pheromone Biosynthesis in *Helicoverpa armigera*

**DOI:** 10.3389/fphys.2021.707389

**Published:** 2021-08-04

**Authors:** Shuangyan Yao, Yunhui Zhang, Yanpeng Chang, Xiang Li, Wenli Zhao, Shiheng An

**Affiliations:** State Key Laboratory of Wheat and Maize Crop Science, College of Plant Protection, Henan Agricultural University, Zhengzhou, China

**Keywords:** HaPYK, acetyl-CoA, Z11-16: Ald, mating behavior, PKC, sugar feeding

## Abstract

Pyruvate kinase (PYK) is a speed-limited enzyme of glycolysis that catalyzes the formation of pyruvate, and plays an important role in acetyl-CoA synthesis. The acetyl-CoA is the precursor of sex pheromone biosynthesis in *Helicoverpa armigera*. However, the role of PYK in sex pheromone biosynthesis remains elusive. Here, *PYK* in *H. armigera* (*HaPYK*) was found to be highly expressed in the pheromone glands (PGs). The developmental expression profile of *HaPYK* was consistent with the fluctuation of sex pheromone release. Function analysis revealed that the knockdown of *HaPYK* led to a decrease in the levels of pyruvic acid and acetyl-CoA in PGs, which in turn caused a significant decrease in cis-11-hexadecenal (Z11-16: Ald) production, female capability to attract males, and mating frequency. Further study demonstrated that sugar feeding (5% sugar) increased the transcription and enzyme activity of HaPYK, thereby facilitating sex pheromone biosynthesis. Moreover, pheromone biosynthesis activating neuropeptide (PBAN) upregulated HaPYK activity through protein kinase C (PKC), as shown by PKC-specific inhibitor analysis. Altogether, our results revealed that PBAN activated HaPYK by Ca^2+^/PKC, thereby regulating the synthesis of pyruvate and subsequent acetyl-CoA, ensuring the supply of sex pheromone precursor, and finally facilitating sex pheromone biosynthesis and mating behavior.

## Introduction

Powerful reproduction capacity is an important reason why insects are the most prosperous species on earth. Accordingly, insects have evolved various ways to communicate to ensure the normal mating and reproduction. Lepidopteran female moths usually use sex pheromone as chemical signal for long-distance communication. After a male moth detects the sex pheromone, it accurately locates and then triggers a series of subsequent courtship and mating behaviors with the female (Rafaeli and Jurenka, 2003; Zhao et al., 2018). Sex pheromone compounds are divided into type I, type II, and miscellaneous (Ando et al., 2004). Type I and type II pheromones are defined by the presence and absence of a terminal functional group, respectively. Type I sex pheromones are characterized by a C10–C18 straight chain with zero to three double bonds at various positions and with different configurations (Ando et al., 2004). Usually, typical type I compounds have a functional group at the terminal position, such as alcohol, ester, or aldehyde functional groups (Wei et al., 2006). Typical type-I compounds are biosynthesized in insect species belonging to the families of Bombycidae, Noctuidae, Sesiidae, Tortricidae, and several others. By contrast, type II sex pheromones are typically generated from insect species belonging to Erebidae, Geometridae, and Tischeriidae (Ando and Yamamoto, 2019). Type II compounds contain a C17–C25 straight chain with polyunsaturated two to five double bonds and corresponding epoxy structure (Millar, 2000, 2010; Wei et al., 2006). In addition to the differences in chemical structure, type I pheromones are also different from type II pheromones in terms of biosynthetic pathways. Most type-I pheromones are biosynthesized from acetyl-CoA via fatty acid biosynthesis followed by the modification of carbon chain such as desaturation, reduction, and acetylation (Ando et al., 2004). In contrast, most type II pheromones are biosynthesized in the oenocytes from dietary essential fatty acids via carbon chain elongation, decarboxylation, and subsequent optional epoxidation (Rule and Roelofs, 1989; Millar, 2000, 2010; Wei et al., 2003, 2004).

For most moths, studies have shown that a novel neuropeptide, pheromone biosynthesis activating neuropeptide (PBAN), regulates sex pheromone biosynthesis. PBAN is produced in the subesophageal ganglion and directly acts on the pheromone glands (PGs) of female moths to initiate sex pheromone biosynthesis (Tillman et al., 1999). The molecular mechanism underlying PBAN-regulated sex pheromone biosynthesis has been extensively studied after PBAN was first identified in *Heliothis zea* females (Raina et al., 1989). Generally, studies on the roles of PBAN mainly focused on two typical species, *Bombyx mori* and *Helicoverpa armigera*. In *B. mori*, PBAN interacts with its receptor localized at the membrane of PG cells and triggers an influx of extracellular Ca^2+^, which in turn activates both calcineurin (CaN) and calmodulin-dependent kinase II (CamKII). CaN is thought to activate fatty acid reductase, whereas CamKII phosphorylates lipid storage droplet protein, thereby promoting the lipolytic release of pheromone precursors, thereby facilitating sex pheromone biosynthesis (Jurenka, 2017). By contrast, in *H. armigera*, PBAN employs Ca^2+^ and cAMP as second messengers when it binds with its receptor. The influx of Ca^2+^ activates CaM to form the CaM/CaN complex. The activated CaN in turn activates the rate-limiting enzyme of fatty acid biosynthesis, namely, acetyl CoA-carboxylase (ACC), by dephosphorylation, thereby promoting the biosynthesis of sex pheromone. The cAMP/PKA signal indirectly activates ACC by inhibiting adenosine 5′-monophosphate (AMP)-activated protein kinase (AMPK) to ensure the biosynthesis of sex pheromone in this species (Du et al., 2017a). These studies deepen our understanding of how PBAN mediates the biosynthesis of species-specific sex pheromones.

Although PBAN-regulated biosynthesis of sex pheromone differs in various moths, most moths employ acetyl-CoA as a precursor of type I sex pheromone to produce sex pheromone through the fatty acid biosynthesis process, followed by the modification of desaturation and reduction (Rafaeli and Jurenka, 2003). As an important metabolite, acetyl-CoA usually originates from glucose, fatty acid, and amino acid catabolism. In the glycolysis process, glucose is transformed into cytosolic pyruvate, which is imported into the mitochondria to form acetyl-CoA through the pyruvate dehydrogenase complex (Herzig et al., 2012). In addition, acetyl-CoA is also generated through the β-oxidation of fatty acids (Morse and Meighen, 1987). Studies have demonstrated that in moths sex pheromone is biosynthesized by using hemolymph trehalose. In *Heliothis virescens*, hemolymph trehalose that originates from sugar feeding is used to generate acetyl-CoA via glycolysis and tricarboxylic acid cycle, which ultimately contributes to sex pheromone biosynthesis through fatty acid biosynthesis (Foster, 2009). Even most of the production of sex pheromone originates from a single feeding of moth (Foster and Anderson, 2015). Similarly, in *Mythimna separata*, sugar feeding markedly increases the concentrations of trehalose, which in turn increases pyruvic acid and acetyl-CoA production in PGs, thereby ultimately facilitating sex pheromone biosynthesis (Zhang et al., 2020). These results revealed that the importance of hemolymph trehalose to sex pheromone biosynthesis originated from supplementary nutrition. Even in some moths that lack supplementary nutrition, acetyl-CoA is required for sex pheromone precursor. For example, in *B. mori*, fatty acids are biosynthesized via acetyl-CoA and then stored in PG cells in the form of triacylglycerols (TAG). PBAN promotes TAG cleavage and fatty acid reduction by regulating the activities of lipase and fatty acid reductase, and ultimately regulating the biosynthesis of sex pheromone (Ohnishi et al., 2006). These studies indicate that acetyl-CoA is a crucial precursor for sex pheromone biosynthesis.

Glycolysis is vital for sex pheromone biosynthesis in most moths. As the rate-limiting step in glycolysis, pyruvate kinase (PYK) catalyzes phosphoenolpyruvate to generate pyruvic acid. The role of PYK in regulating glycolysis has been extensively studied in vertebrates and invertebrates (Hall and Cottam, 1978; Fang et al., 1992). As reported, PKC directly controls the activity of PYK (Dailianis and Kaloyianni, 2004; Kaloyianni et al., 2004). However, the detailed mechanism underlying role of PYK in sex pheromone biosynthesis in moths remains elusive. In the present study, *H. armigera* was chosen as a model to investigate the role of PYK in PBAN-regulated sex pheromone biosynthesis. The results of the present study provide foundational evidence for the function of carbohydrate metabolism enzyme in sex pheromone biosynthesis and help screen new targets for pest control.

## Materials and Methods

### Materials

#### Insects

*H. armigera* larvae were reared by artificial medium in our laboratory with 26 ± 1°C temperature, 75 ± 1% humidity, and 14L:10D photoperiod (Zhao et al., 2016).

#### Chemicals

PBAN (Ser-Arg-Thr-Lys-Tyr-Phe-Ser-Pro-Arg-Leu-NH_2_) was synthesized from the Sangon Company (Sangon, Shanghai, China). Z11-hexadecenal (Z11–16:Ald) was obtained from the Sigma Company (Sigma, St. Louis, USA) and used to determine sex pheromone titer by gas chromatography/mass spectrometry (GC-MS), according to previous description (Zhao et al., 2018).

### Methods

#### Double-Stranded RNA Synthesis and RNA Interference

The double-stranded RNA (dsRNA) was generated by MEGAscript RNA interference (RNAi kit; Thermo, Waltham, USA) following the instructions of the manufacturer. Firstly, DNA template for ds*HaPYK* was amplified by PCR (716 bp) with PG cDNA and specific primers containing T7 promoter sequence (*HaPYK*-T7F: GATCACTAATACGACTCACTATAGGGAGAGAATGGAGGTATGCTGGGTT; *HaPYK*- T7R: GATCACTAATACGACTCACTATAGGGAGAGGACTTGCCGCTTGTGGT). The dsRNA was synthesized *in vitro* by using MEGAscript RNAi kit according to previous description (Du et al., 2012). In order to remove template DNA and single-stranded RNA, DNase and RNase were added to the dsRNA reaction system followed by purification by MEGAclear™ column and elution with diethyl pyrocarbonate-treated nuclease-free water. The enhanced green fluorescent protein (EGFP; GenBank accession number MN623123.1, 538 bp) dsRNA was used as a negative control (*EGFP*-T7F: GATCACTAATACGACTCACTATAGGGAGACACAAGTTCAGCGTGTCCG; *EGFP*-T7R: GATCACTAATACGACTCACTATAGGGAGAGTTCACCTTGATGCCGTTC). The dsRNA quality was checked by a biophotometer (Eppendorf) and agarose gel electrophoresis.

Newly-emerged females were firstly decapitated and then injected with 10 μg ds*HaPYK* at the intersegmental membrane between the eighth and ninth abdominal segments. PGs were then collected at 48 h after dsRNA injection. Females injected with 10 μg ds*EGFP* were used as the control. The RNAi efficiency was tested by qRT-PCR. Three biological replicates were employed for ensuring experiment reliability and repeatability, and every replicate contained at least 15 females.

#### qRT-PCR

Total RNA was extracted using Trizol reagent following to the instructions of the manufacturer. Purity and integrity of the extracted RNA was analyzed by agarose gel electrophoresis and RNA Nano 6000 Assay Kit with the Agilent Bioanalyzer (Agilent, Santa Clara, USA). The cDNA was synthesized by HiScript III RT SuperMix kit (+ gDNA cursor) (Vazyme, Nanjing, China) and used as qRT-PCR template. The *18s* gene was employed as the internal reference gene (*18S*-RTF: GCATCTTTCAAATGTCTGC; *18S*-RTR: TACTCATTCCGATTACGAG) (Du et al., 2017a). The real-time quantitative PCR of *HaPYK* (*HaPYK*-RTF: AAGATGCTGGTTACCGAATGT; *HaPYK*-RTR: AAGGGACCAGGTAAAGACAT) was performed on Applied Biosysterms 7500 fast real-time PCR instrument (ABI, Carlsbad, USA) by using ChamQ Universal SYBR qPCR Master Mix (Vazyme, Nanjing, China). The qPCR conditions were as follows: 95°C for 5 min, followed by 40 cycles of 95°C for 15 s and 60°C for 20 s. The melting curve and agarose gel electrophoresis were used to confirm the specificity of the PCR signal. The comparative cross threshold method (CT, the PCR cycle number that crossed the signal threshold) was used to quantify the relative expression level (Livak and Schmittgen, 2001). Three biological replicates and three technical repetitions were used to ensure the reliability and reproducibility of qRT-PCR results. The significant differences in the expression of each gene of different treatments were compared with Student's *t*-test.

#### Sugar Feeding

Newly-emerged females were collected and put into cage (30 × 30 × 30 cm) with 5% sugar. PGs were collected after different time points of feeding (24, 48, and 72 h) and subjected to subsequent analysis of qRT-PCR, enzyme activity, and Z11-16:Ald level. Females fed with water were chosen as control (Liang et al., 1999).

#### Measurement of Z11-16: Ald Production

The effect of *HaPYK* knockdown on sex pheromone production was investigated according to method previously described (Zhao et al., 2018). Briefly, newly-emerged females were decapitated and injected with 10 μg ds*HaPYK*. About 48 h after injection, the treated females were further injected with PBAN (10 pM) for 1 h. PGs were then collected at 1 h after PBAN injection and dissolved in hexane for GC-MS determination as described previously (Zhao et al., 2018). The GC conditions were as follows: injection at 60°C and programed to 250°C at 5°C/min, and then to 280°C at 10 min. Sex pheromone components (Z11-16: Ald) were identified by the retention time and mass spectra with standard sample of Z11-16: Ald. Females injected with equal qualities of ds*EGFP* were employed as a negative control.

The effect of sugar feeding on Z11-16: Ald production was investigated. Briefly, newly-emerged females were collected and put into cage (30 × 30 × 30 cm) with 5% sugar. PGs were collected after different time points of feeding (24, 48, and 72 h) and dissolved in hexane for GC-MS determination. Three biological replicates were employed and each biological replicate contains at least 20 PGs.

#### The Measurement of Pyruvic Acid and Acetyl-CoA Contents

The effect of PBAN on pyruvic acid was investigated. Briefly, PGs were harvested from 48 h-old females and incubated with Graces' medium for 2 h. The medium was replaced by new Graces' medium with 10 pM PBAN. PGs were collected at different treatment times of PBAN (0, 10, 30, 60, and 90 min) and subjected to measurement of pyruvic acid content by using pyruvate acid assay kit (Solarbio, Beijing, China) according to the instructions of the manufacturer. The experiment was performed with three biological replicates, and every replicate contained at least 30 PGs.

The effect of *HaPYK* knockdown on pyruvic acid production was investigated. Briefly, newly-emerged females were injected with 10 μg ds*HaPYK* 24 h after injection, and PGs were harvested and added to the extract buffer, which was provided by pyruvate acid assay kit (Solarbio, Beijing, China) at a ratio of 1:10. PGs were homogenized in ice and incubated in ice for 30 min. After centrifugation at 8,000*g* for 1 min, the supernatant was obtained. The supernatant was added to the reaction solution, and OD values at 520 nm were recorded to calculate the pyruvate content (Fu et al., 2019). Three biological replicates were employed, and every biological replicate contained at least 30 PGs. The females injected with ds*EGFP* were chosen as control.

The effect of *HaPYK* knockdown on acetyl-CoA content was investigated. Briefly, newly-emerged females were injected with 10 μg ds*HaPYK* and 24 h after injection, PGs were collected and homogenized on ice. The homogenized mixture was centrifuged at 8,000 rpm for 4 min. The supernatant was obtained to determine the content of acetyl-CoA according to method described in acetyl-CoA content kit (Jiancheng, Nanjing, China). The OD values at 340 nm of supernatant reaction were used to calculate the content of acetyl-CoA. The control females were injected with ds*EGFP* (10 μg). Three biological replicates were employed and every biological replicate contained at least 30 PGs.

#### Mating Behavior

The newly-emerged females were firstly injected with 10 μg ds*HaPYK* and 24 h after injection, the treated females were put into a cage (60 × 60 × 60 cm). The same number of males were put into the cage. After waiting 24 h, mating proportion of females was calculated according to presence of spermatophore in female bursa copulatrix, following the previous method (Du et al., 2017b). Three biological replicates were employed to ensure the reliability and repeatability of the experiments, and every replicate contained at least 15 females. The females injected with ds*EGFP* were employed as negative control.

#### Female Ability to Attract Male

The female ability to attract males was investigated according previous report (Du et al., 2017b). Briefly, newly emerged females (*n* = 15) were injected with 10 ug ds*HaPYK* or 10 ug ds*EGFP*. After 48 h of dsRNA injection, treated females were placed in a trap cell (28 cm high × 30 cm wide × 30 cm long). The control females injected with 10 ug ds*EGFP* were placed into another trap cell. Two-day-old males (*n* = 50) were placed into released cell (height 32 cm × width 30 cm × length 60 cm) located at the upper part of trap cell. After 24 h, the number of males in each trap cell was recorded.

#### The Measurement of HaPYK Activity

The PGs (*n* = 30) were collected from 2-day old females and then incubated in Graces' medium for 2 h. The medium was replaced by a new medium with PBAN (10 pM). PGs were collected at different time points of PBAN treatments (0, 10, 30, 60, and 90 min) and subjected to HaPYK activity analysis using the PYK activity kit from the Abcam company (Abcam, Cambridge, UK) following the instructions (Zhang et al., 2019). Three biological replicates were employed and every replicate contained at least 30 PGs.

PG were harvested from 2-day old females and then incubated in Graces' medium for 2 h. The medium was replaced by a new medium with PKA inhibitor H-89 (20 μM) or PKC inhibitor chelerythrine chloride (CC) (10 μM). One hour after incubation of H-89 (20 μM) or CC (10 μM), the medium 10 pM PBAN was added. PGs were collected at different time points of PBAN treatments (0, 10, 30, 60, and 90 min) and subjected to PYK activity analysis using the PYK activity kit from the Abcam company (Abcam, Cambridge, UK) following to instruction (Zhang et al., 2019). Three biological replicates were employed and every replicate contained at least 30 PGs.

## Results

### The Developmental and Spatial Expression Profiles of *HaPYK*

**Figure 1:**
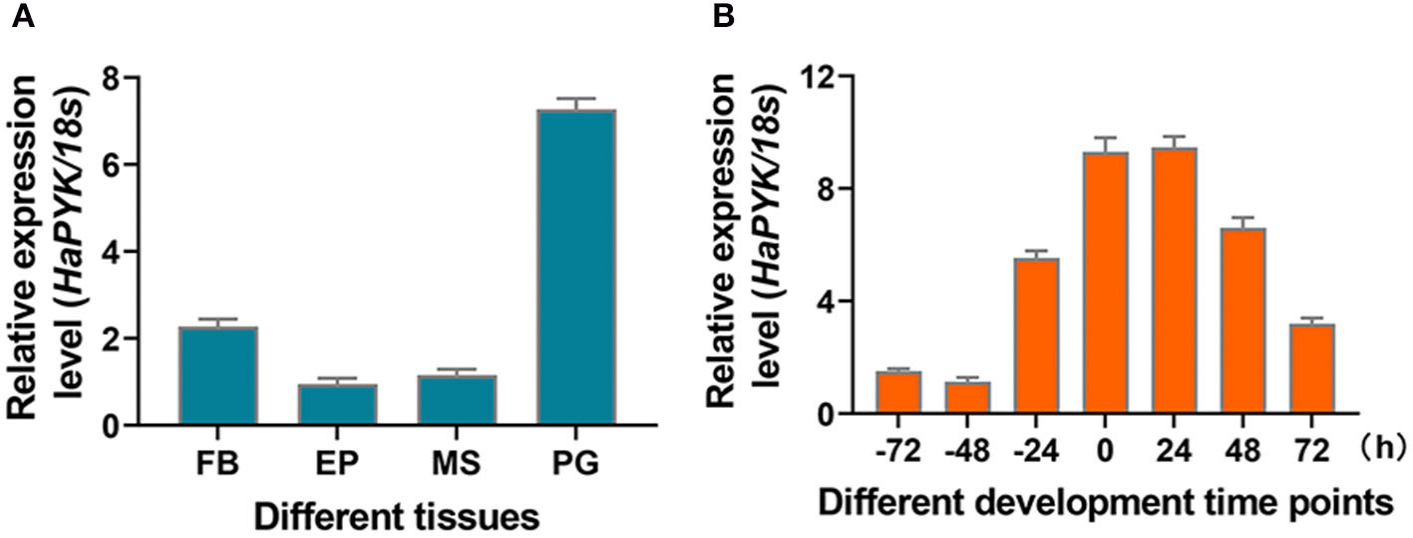
The expression profile of *HaPYK*. **(A)** The tissue distribution of *HaPYK* by qRT-PCR. FB, fat body; EP, epidermis; MS, muscle; PG, pheromone gland. **(B)** The expression profile of *HaPYK* in the PGs by qRT-PCR. Newly-emerged adult was designated as 0 h-old adult. The *18S* was used as the internal reference. Error bars indicated the mean ± s.d. of three independent biological experiments and three technical repetitions.

The tissue distribution of *HaPYK* manifested that *HaPYK* was expressed in all the examined tissues (fat body, epidermis, flight muscle, and PGs), and the highest expression level was in the PGs (Figure 1A). The developmental expression pattern of *HaPYK* in the PGs demonstrated that *HaPYK* transcript was detected 72 h before emergence, and continued to increase from 24 h before emergence to 24 h after emergence and peaked at 24 h after emergence (Figure 1B). The expression pattern of *HaPYK* in the PGs was consistent with the fluctuation of sex pheromone release, indicating its potential role in sex pheromone biosynthesis (Du et al., 2017a).

### *HaPYK* Knockdown Decreased the Contents of Pyruvic Acid and Acetyl-CoA in the PGs

**Figure 2:**
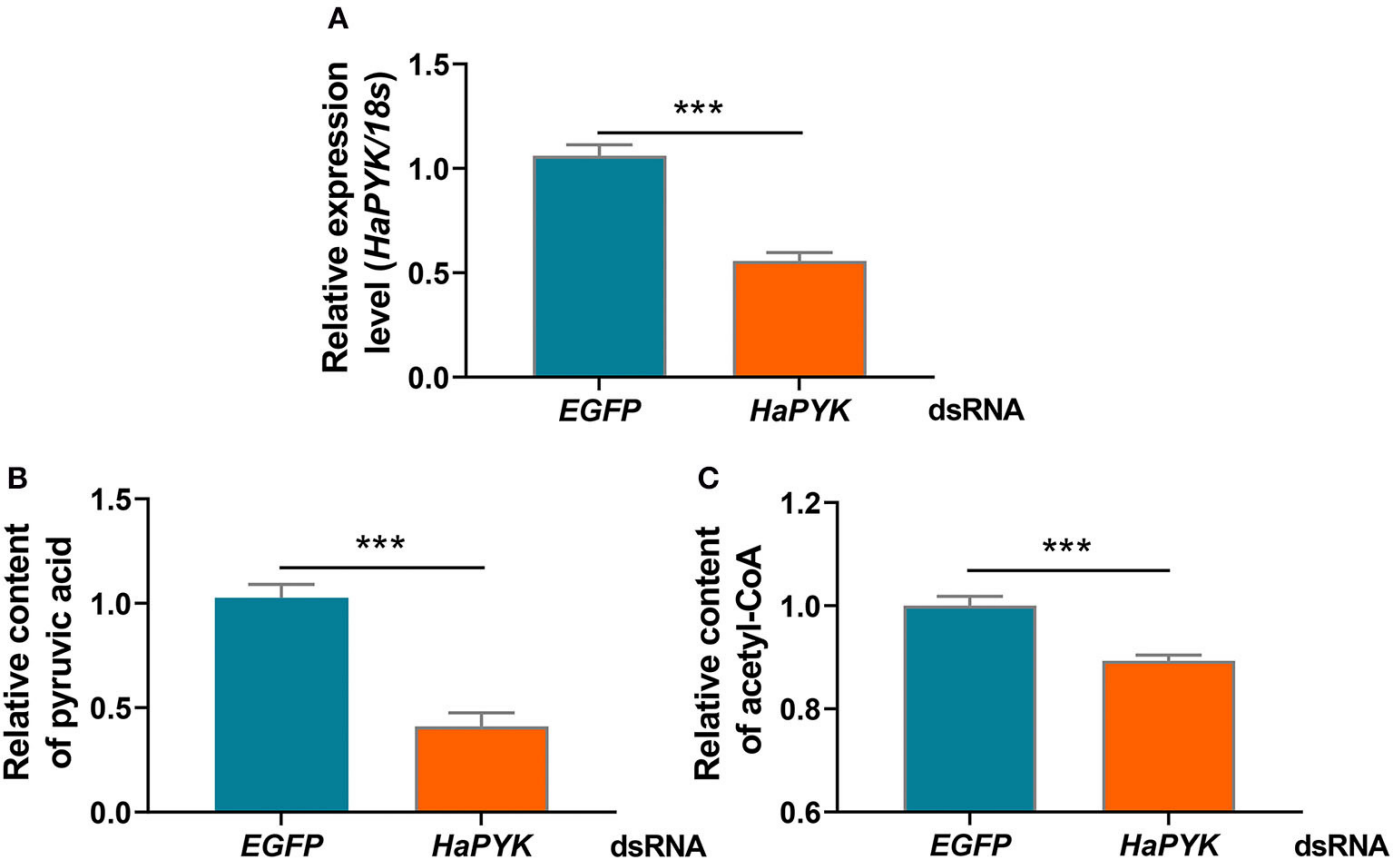
Knockdown of *HaPYK* decreased the contents of pyruvic acid and acetyl-coA. **(A)** Inference efficacy of *HaPYK* in PGs. **(B,C)** The effect of *HaPYK* knockdown on the contents of pyruvic acid and acetyl-coA. Error bars indicated the mean ± s.d. of three independent biological experiments, ^***^P < 0.001 (Student's *t*-test).

RNAi was employed to investigate the effect of *HaPYK* knockdown on the contents of pyruvic acid and acetyl-CoA in PGs. Results demonstrated that the injection of *dsHaPYK* in females led to a significant decrease in the expression of *HaPYK* transcript, compared with those in females injected with *dsEGFP* (Figure 2A). After successful knockdown of *HaPYK*, the effects of *HaPYK* knockdown on the contents of pyruvic acid and acetyl-CoA in PGs were further investigated, and the results manifested that RNAi-mediated knockdown of *HaPYK* caused to a significant decrease in pyruvic acid and acetyl-CoA production in PGs (Figures 2B,C).

### *HaPYK* Knockdown Decreased the Sex Pheromone Production and Subsequent Mating Behaviors

**Figure 3:**
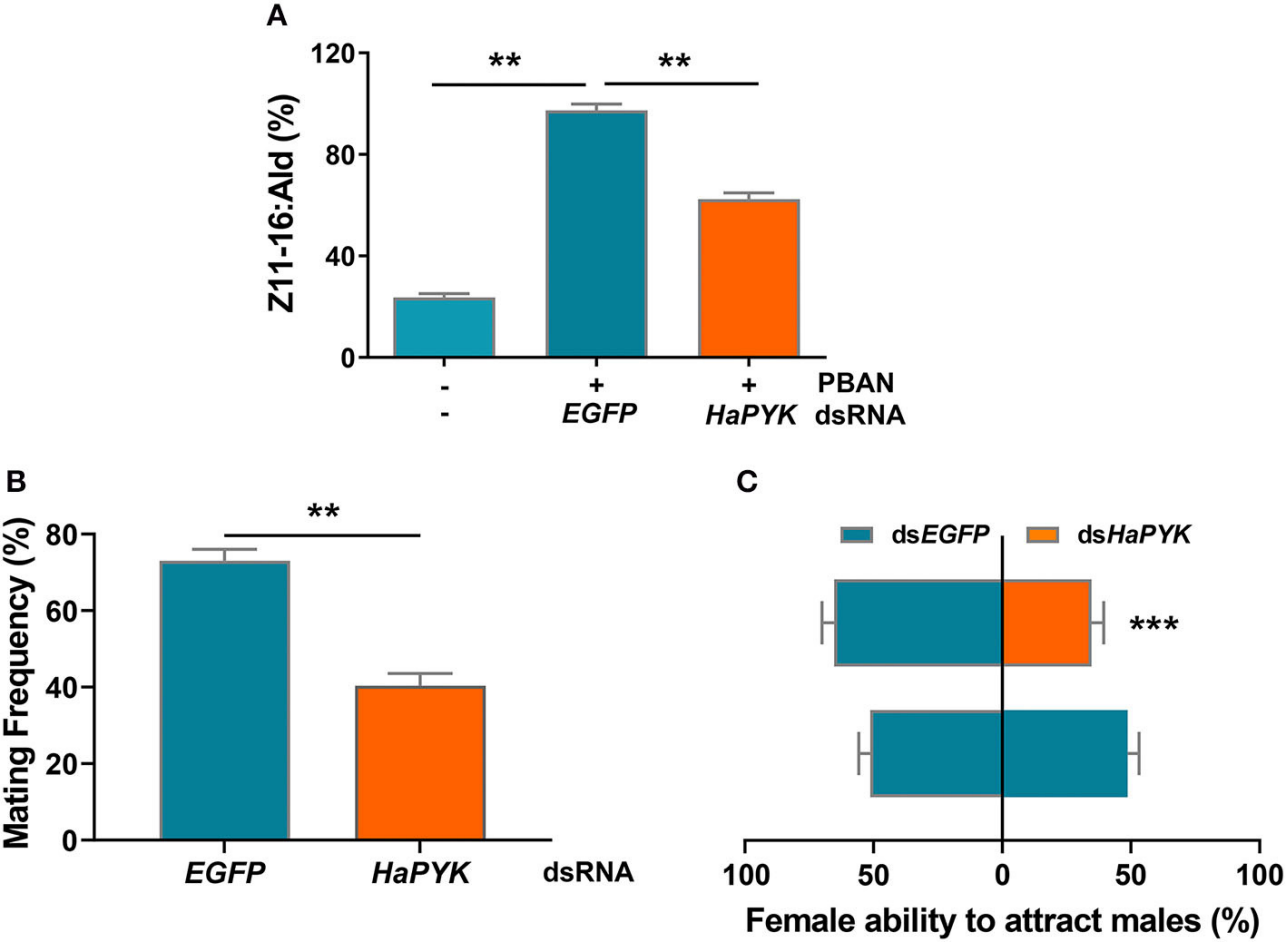
Knockdown of *HaPYK* reduced the content of sex pheromone and mating behaviors. **(A)** The effect of *HaPYK* knockdown on sex pheromone production. **(B,C)** The effect of *HaPYK* knockdown on mating frequency and attractant ability. Error bars indicate the mean ± s.d. of three independent biological experiments, ^**^P < 0.01, ^***^P < 0.001 (Student's *t*-test).

The effects of *HaPYK* knockdown on sex pheromone production and subsequent mating behaviors were further investigated, and the results showed that RNAi-mediated knockdown of *HaPYK* significantly reduced the production of sex pheromone regulated by PBAN, as shown by GS/MS analysis, compared with the controls that were injected with ds*EGFP* (Figure 3A). Accordingly, the decrease of sex pheromone production caused by ds*HaPYK* injection in turn led to a significant decrease in female ability to attract males and mating frequency, compared with the controls that were injected with ds*EGFP* (Figures 3B,C). These results indicated that *HaPYK* was required for PBAN-regulated sex pheromone biosynthesis and subsequent mating behaviors.

### PBAN Activated HaPYK Activity via PKC

**Figure 4:**
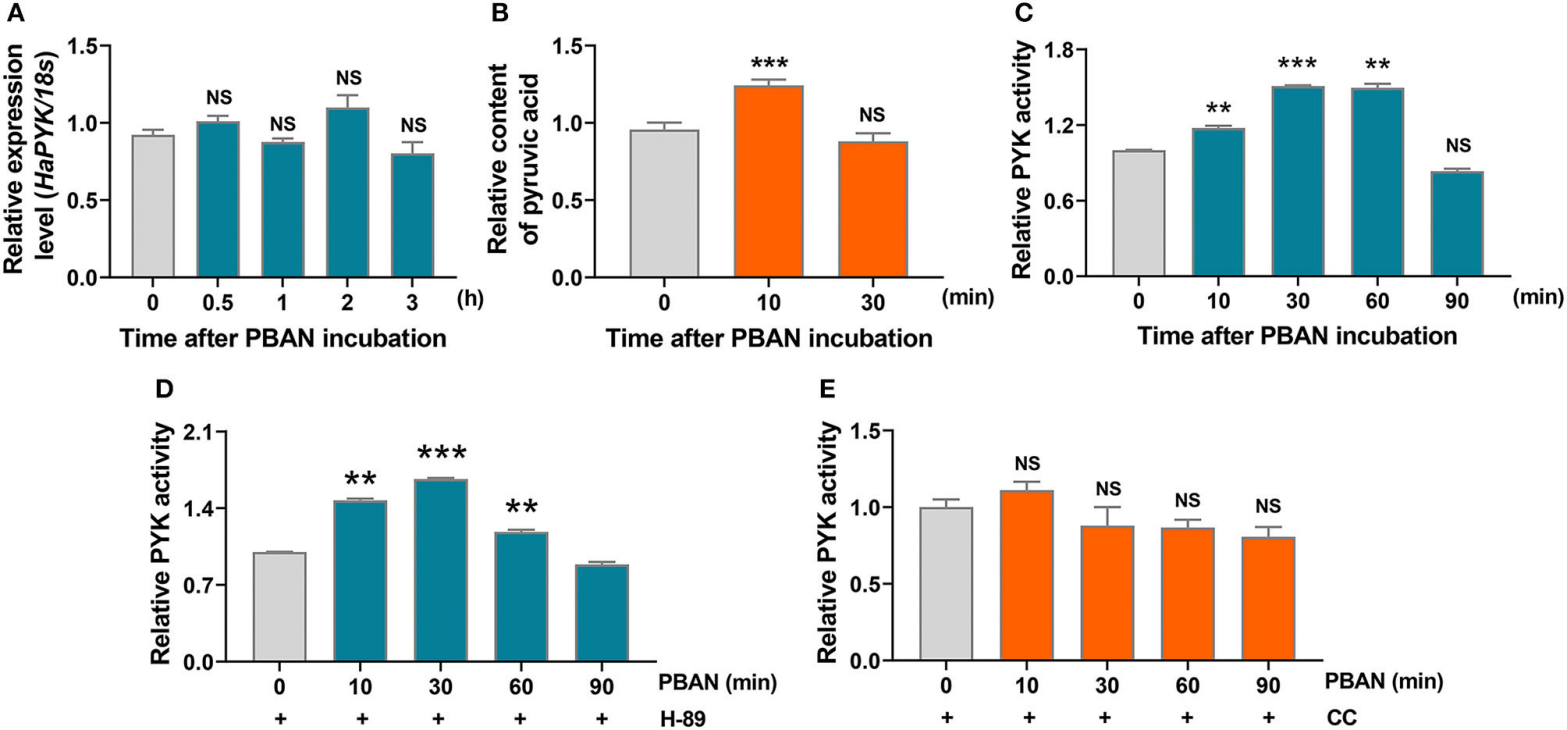
PBAN regulated HaPYK activity through PKC. **(A)** The effect of PBAN treatment on the mRNA level expression of *HaPYK*. 18S was the internal reference. Error bars indicate the mean ± s.d. of three independent biological experiments. **(B)** The effect of PBAN treatment on the content of pyruvic acid. Error bars indicate the mean ± s.d. of three independent biological experiments, ^***^P<0.001 (Student's *t*-test). **(C)** The effect of PBAN treatment on HaPYK activity. Error bars indicated the mean ± s.d. of three independent biological experiments, ^**^P < 0.01, ^**^P < 0.001(Student's *t*-test). **(D)** The effect of PKC inhibitor (CC) on HaPYK activity. Error bars indicate the mean ± s.d. of three independent biological experiments. **(E)** The effect of PKA inhibitor (H-89) on HaPYK activity. Error bars indicated the mean ± s.d. of three independent biological experiments, ^**^P < 0.01, ^***^P < 0.001(Student's *t*-test).

The effect of PBAN on HaPYK activity was investigated, and the result demonstrated that PBAN treatment had no effect on the mRNA expression of *HaPYK* (Figure 4A), indicating that PBAN did not regulate the expression of *HaPYK* in mRNA level. Interestingly, PBAN treatment significantly increased the content of pyruvic acid (Figure 4B), indicating that PBAN indeed mediated the glycolysis process to generate pyruvic acid. Further study confirmed that PBAN treatment significantly increased HaPYK activity (Figure 4C), which was consistent with the result that PBAN-regulated the biosynthesis of pyruvic acid. H-89 and CC treatment further revealed that inhibition of PKA activity did not prevent the PBAN-induced increase of HaPYK activity (Figure 4D). However, the inhibition of PKC activity with CC significantly attenuated the PBAN-induced increase of HaPYK activity (Figure 4E). These results revealed that the PBAN-regulated increase of HaPYK activity was dependent on PKC signaling.

### Sugar Feeding Promoted the Increase of HaPYK Activity and Z11-16:Ald Content in the PGs

**Figure 5:**
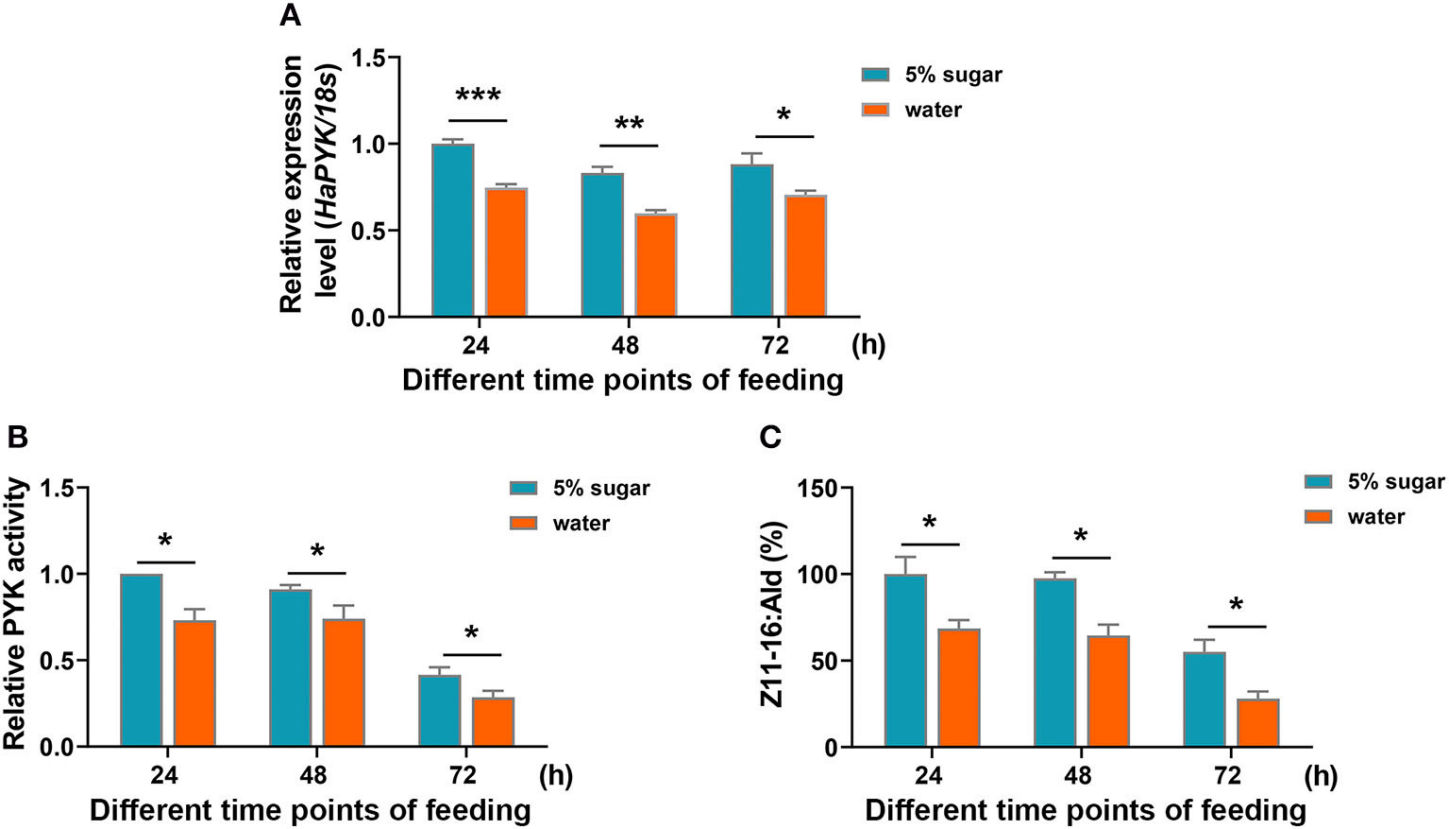
Sugar feeding increased HaPYK activity and subsequent Z11-16:Ald production. **(A)** The expression analysis of *HaPYK* in PGs under sugar/water feeding for different time points (24, 48, and 72 h) by qRT-PCR. The 18S was used as the internal reference. Error bars indicated the mean ± s.d. of three independent biological experiments and three technical repetitions. ^*^P < 0.05, ^**^P < 0.01, ^***^P < 0.001(Student's *t*-test). **(B)** The effect of sugar feeding on HaPYK activity. Error bars indicated the mean ± s.d. of three independent biological experiments and three technical repetitions. ^*^P < 0.05 (Student's *t*-test). **(C)** The effect of sugar feeding on Z11-16:Ald production in PGs. Error bars indicated the mean ± s.d. of three independent biological experiments and three technical repetitions. ^*^P < 0.05 (Student's *t*-test).

Sugar feeding (5% sugar) significantly induced the transcription of *HaPYK* compared with the controls that were fed with water (Figure 5A). Correspondingly, HaPYK activity also significantly increased after 5% sugar feeding (Figure 5B), thereby indicating that HaPYK responded to the feeding signal to increase the expression of *HaPYK* mRNA level and HaPYK activity. Further GC-MS analysis demonstrated that sugar feeding facilitated to Z11-16:Ald biosynthesis in the PGs when compared with females fed with water (Figure 5C). These results revealed that sugar feeding promoted the transcriptional and enzyme activities of HaPYK, and finally facilitated to sex pheromone biosynthesis.

## Discussion

Most adult Lepidoptera feed exclusively on nectar containing water and sugar. This habit in the adult stage contributes to the increase in fecundity. For example, *Spodoptera exempta* adults lay fewer eggs and live for a shorter duration when carbohydrate is absent in their diet. However, sucrose supplement rescues their reproductive potential (Gunn et al., 1989). In *H. zea*, females began to lay eggs under the condition of only water, but fecundity increased by 2-folds when sucrose supply became available (Lukefahr and Martin, 1964). In *H. virescens* (Fabricius) and *Alabama argillacea* (Hiibner), compared with females fed with water, sucrose feeding could increase the fecundity five times (Lukefahr and Martin, 1964). These results indicated that supplemental nutrition on the adult stage increases female fecundity. Not only that, sugar feeding in females also increases sex pheromone production. For instance, in female *H. virescens*, sugar feeding increases hamolymph trehalose concentration, which probably influences glycolysis in gland cells, thereby affecting the levels of acetyl-CoA and ultimately mediating sex pheromone production (Foster, 2009). Similarly, in female *M. separata*, sugar feeding facilitates the increase in the concentrations of trehalose, pyruvic acid, and acetyl-CoA in PGs, thereby resulting in the significant increase of sex pheromone titer, female ability to attract males, and successful mating frequency (Zhang et al., 2020). These results elucidated the importance of supplemental nutrition for sex pheromone biosynthesis in these moths. In fact, this phenomenon was not difficult to understand because the nectar intake can rapidly enter the glycolysis process, thereby providing the essential materials (acetyl-CoA) for sex pheromone biosynthesis. Surprisingly, sugar intake plays such an important role in sex pheromone biosynthesis. For example, studies have showed that 65% of sex pheromone production of female moths comes from a single feeding (Foster and Anderson, 2015). These studies hint that glycolysis plays an important role in biosynthesizing the sex pheromone precursor, acetyl-CoA. However, the mechanism underlying the role of glycolysis in sex pheromone biosynthesis is not well-studied. Thus, we focused on a speed-limited enzyme of glycolysis, PYK, and presented new insights on the role of PYK in PBAN-mediated sex pheromone biosynthesis.

PYK catalyzes the irreversible transphosphorylation from phosphoenolpyruvate and ADP to pyruvate. Pyruvate is the final product of the glycolysis pathway. It can be reduced to lactic acid in the cytoplasm for energy supply. It can be oxidized into acetyl-CoA in the mitochondria and enters the tricarboxylic acid cycle. It is then is oxidized into carbon dioxide and water to complete the aerobic oxidation of glucose for energy supply. Most importantly, pyruvate can also achieve the mutual conversion of sugars, fat, and amino acids in the body through the acetyl-CoA and tricarboxylic acid cycles. Therefore, pyruvate plays an important role in the metabolic linkage of the three nutrients (Melkonian and Schury, 2020). The importance of pyruvate in the metabolism of sugars, fat, and amino acids places PYK at a crucial metabolic intersection. The importance of PYK in glycolysis pathway prompted us to investigate role of PYK in sex pheromone biosynthesis. As expected, RNAi-mediated knockdown of HaPYK significantly decreased the contents of pyruvic acid and acetyl-CoA in the PGs, thereby showing a general role of PYK in the process of glycolysis. Correspondingly, the decrease of pyruvic acid and acetyl-CoA contents in turn reduced sex pheromone production, which finally caused a decrease in female ability to attract males and in the mating frequency of females. These results were in agreement with finding obtained for *H. virescens*, where the absence of sugar feeding decreased hemolymph trehalose concentration and the levels of acetyl-CoA, which finally decreased sex pheromone production (Foster, 2009). Similar results were also found in female *M. separata*, in which blocking of the sugar intake led to a decrease of glycolysis efficiency, as shown by the decrease in the concentrations of trehalose, pyruvic acid, and acetyl-CoA in the PGs, which finally resulting in the significant decrease of sex pheromone titer and subsequent mating efficiency (Zhang et al., 2020). These results were also consistent with the process of sex pheromone biosynthesis. As we have known, most sex pheromones are synthesized from fatty acids by using acetyl-CoA, which originated from glycolysis, as the material. Thus, any factor that affects glycolysis will inevitably influence acetyl-CoA and sex pheromone production. This is the reason why changes in some of the factors, including adult supplemental nutrition, PYK, and trehalase finally affected sex pheromone biosynthesis in the present study and in a previous study (Foster, 2009; Zhang et al., 2020). In fact, many moths such as *H. armigera, M. separata*, and *H. virescens* have a habit of feeding on nectar, and usually synthesize sex pheromone by utilizing supplementary carbohydrate. In these moths, the supplementary carbohydrate plays an important role in sex pheromone biosynthesis (Foster and Anderson, 2015). However, adults of other moths, for example, *B. mori*, do not have the habit of obtaining supplementary nutrition (Matsumoto, 2010). They typically utilize the nutrition obtained by the larvae to synthesize fatty acid, and then store fatty acids in PGs in the form of triacylglycerols (TAGs), which act as precursors for sex pheromone (Matsumoto, 2010). Even so, in this kind of moth, glycolysis is essential to produce acetyl-CoA. Thus, HaPYK plays an important role in sex pheromone biosynthesis, although the detailed mechanism needs to be addressed in moths that do not feed during the adult stage.

**Figure 6:**
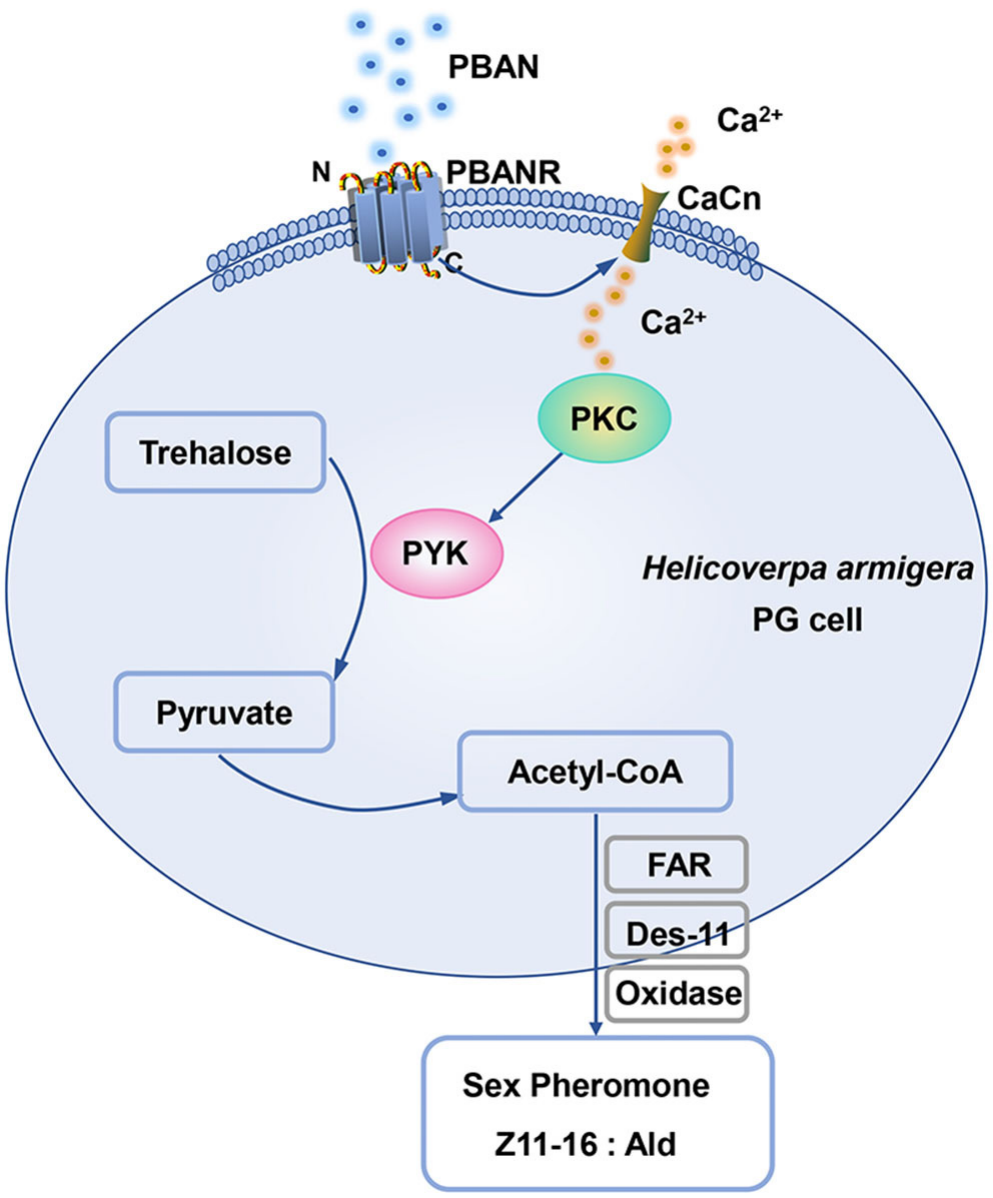
The diagram of HaPYK in the PBAN-induced sex pheromone biosynthesis.

PYK was positively regulated by glucose. Studies confirmed that glucose serves as an important activator to active mRNA level expression of PYK. In human erythrocytes, the addition of glucose significantly enhances PYK activity (Kaloyianni et al., 2004). It is well-known that glucose-mediated activation of PYK transcript is dependent on the activation of the glucose-response element located at the PYK promoter region (Krones et al., 2001). In the present study, we also found that sugar feeding increased the mRNA expression of PYK transcript and PYK activity, consistent with previous results (Krones et al., 2001; Kaloyianni et al., 2004), which indicated the conserved activation of PYK. These results were also consistent with the finding that the supplemental nutrition of female moth promoted the increase of trehalose, pyruvic acid, and acetyl-CoA levels in PGs, and the increase of sex pheromone production in *H. armigera, M. separata*, and *H. virescens* (Foster, 2009; Zhang et al., 2020), which elucidated the importance of the role of PYK in sex pheromone biosynthesis. Our results also demonstrated that PBAN treatment did not influence the mRNA level expression of the *HaPYK* transcript. However, PBAN treatment increased pyruvic acid contents in PG cells, which indicated that PBAN probably regulated HaPYK activity. Further experiment confirmed our hypothesis that PBAN treatment significantly increased HaPYK activity in a very short time (10 min). The rapid activation of HaPYK inspired us to investigate whether HaPYK was activated by a kinase under PBAN treatment. Given that PKA and PKC are important kinases in the biosynthesis of sex pheromone (Hull et al., 2005; Du et al., 2017a), PKA and PKC specific inhibitors (H-89 and CC) were employed to investigate the effects of PKA and PKC on HaPYK activity. Interestingly, the inhibition of PKC with PKC inhibitor CC significantly offset PBAN-induced HaPYK activity. However, PKA inhibitor could not attenuate PBAN-induced HaPYK activity. These results revealed that PKC, and not PKA, activated HaPYK in response to PBAN simulation. In *H. armigera*, PBAN employed two signal pathways (Ca^2+^ and cAMP/PKA) to mediate sex pheromone biosynthesis. Thus, it can be inferred that PBAN regulated HaPYK activity by Ca^2+^/PKC pathway. Similar results were found in *Mytilus galloprovincialis*, in which the PKC activator resulted in a significant rise in PYK activity in the digestive gland (Dailianis and Kaloyianni, 2004). Also, in human erythrocytes, PKC activator stimulated PYK activity. However, PKC inhibitor abolished the effect of glucose on PYK activity, indicating that PKC activated PYK activity (Kaloyianni et al., 2004). What's more, in unfertilized hen's egg, purified PYK was confirmed to be the substrate of protein kinase C (Noda et al., 1986). These results provided strong evidence that PKC regulates PYK activity directly. Although the activation of HaPYK by PBAN needs further study, we could provide a hypothetical model according to the results of our present study. When PBAN binds with its receptor, it employs Ca^2+^ as a secondary messenger to activate PKC. PKC in turn activates HaPYK activity to regulate the synthesis of pyruvic acid and acetyl-CoA in PGs, thereby ensuring the supply of sex pheromone precursor, and finally mediating sex pheromone biosynthesis in *H. armigera* (Figure 6).

## Data Availability Statement

The original contributions presented in the study are included in the article/supplementary material, further inquiries can be directed to the corresponding authors.

## Author Contributions

WZ and SA conceived the experiment. SY, YZ, and YC conducted the experiment. WZ and SY were responsible for data analysis and critical editing of the manuscript. SA was responsible for the acquisition of funds, critical editing, and revising the manuscript. All authors contributed to the article and approved the submitted version.

## Conflict of Interest

The authors declare that the research was conducted in the absence of any commercial or financial relationships that could be construed as a potential conflict of interest.

## Publisher's Note

All claims expressed in this article are solely those of the authors and do not necessarily represent those of their affiliated organizations, or those of the publisher, the editors and the reviewers. Any product that may be evaluated in this article, or claim that may be made by its manufacturer, is not guaranteed or endorsed by the publisher.
